# P359: The consequences after an aesthetic procedure, is it worth it?

**DOI:** 10.1186/2047-2994-2-S1-P359

**Published:** 2013-06-20

**Authors:** M Triviño, M Grande, MJ Torijano, M Terol, C Gil, P Rodríguez

**Affiliations:** 1 Preventive Medicine and Quality Management, General University Hospital Gregorio Marañón, Madrid, Spain

## Introduction

*Burkholderia cepacia* is a gram-negative bacillus belonging to the Pseudomonadaceae family that can cause healthcare associated infections from contaminated disinfectants, medical equipment, prosthetic material and drugs such as anesthetics or urological irrigation fluids.

## Objectives

Describe a case of *B. Cepacia* infection following intramuscular injection of prosthetic material in the gluteal region in the context of an aesthetic intervention in a beauty center.

## Methods

Case description.

## Results

We report the case of a woman who was admitted in early June 2012 to our hospital with fever of unknown origin. In early May 2012, she travelled to her country, Venezuela, for an aesthetic procedure consisting of the intramuscular administration of hyaluronic acid in both glutei. Subsequently it was discovered that the substance administered was methacrylate. In late May she travelled to Cancun, where she suffered a hip trauma without fractures or wounds. In June she came back to Madrid (Spain), where two days after she began with general discomfort. A few days later, she started with fever of unknown origin, and was admitted to the Internal Medicine Department for study, where imaging tests reveiled phlegmonous collections and granulomatous inflammatory reaction signs in both gluteal regions. She received antibiotic therapy and several surgical drainages, isolating *B. Cepacia.* Finally, she was discharged in August 2013. This event was reported to the Spanish Agency of Medicines and Health Products.

## Conclusion

data suggest that infection was due to the use of contaminated methacrylate. We were not able to find out if contamination was caused during fabrication or by an unsafe manipulation. Aesthetic treatments should be performed in specialized centers that meet minimum quality security conditions. Moreover, in a situation of potential harm to the health of the population, communication to competent authorities is essential for the implementation of control measures.

## Disclosure of interest

None declared.

